# Genetic Diversity in Cytokines Associated with Immune Variation and Resistance to Multiple Pathogens in a Natural Rodent Population

**DOI:** 10.1371/journal.pgen.1002343

**Published:** 2011-10-20

**Authors:** Andrew K. Turner, Mike Begon, Joseph A. Jackson, Janette E. Bradley, Steve Paterson

**Affiliations:** 1 Institute of Integrative Biology, Biosciences Building, University of Liverpool, Liverpool, United Kingdom; 2 Institute of Biological, Environmental, and Rural Sciences, Aberystwyth University, Aberystwyth, United Kingdom; 3 School of Biology, University of Nottingham, Nottingham, United Kingdom; University of Wisconsin–Madison, United States of America

## Abstract

Pathogens are believed to drive genetic diversity at host loci involved in immunity to infectious disease. To date, studies exploring the genetic basis of pathogen resistance in the wild have focussed almost exclusively on genes of the Major Histocompatibility Complex (MHC); the role of genetic variation elsewhere in the genome as a basis for variation in pathogen resistance has rarely been explored in natural populations. Cytokines are signalling molecules with a role in many immunological and physiological processes. Here we use a natural population of field voles (*Microtus agrestis*) to examine how genetic diversity at a suite of cytokine and other immune loci impacts the immune response phenotype and resistance to several endemic pathogen species. By using linear models to first control for a range of non-genetic factors, we demonstrate strong effects of genetic variation at cytokine loci both on host immunological parameters and on resistance to multiple pathogens. These effects were primarily localized to three cytokine genes (*Interleukin 1 beta* (*Il1b*), *Il2*, and *Il12b*), rather than to other cytokines tested, or to membrane-bound, non-cytokine immune loci. The observed genetic effects were as great as for other intrinsic factors such as sex and body weight. Our results demonstrate that genetic diversity at cytokine loci is a novel and important source of individual variation in immune function and pathogen resistance in natural populations. The products of these loci are therefore likely to affect interactions between pathogens and help determine survival and reproductive success in natural populations. Our study also highlights the utility of wild rodents as a model of ecological immunology, to better understand the causes and consequences of variation in immune function in natural populations including humans.

## Author Summary

Much of what we know about the genetic basis of immunity to infection has come from studies of laboratory animals. However, these animals are kept in conditions very different from those experienced in the natural environment. In order to improve our understanding of the genetic determinants of disease susceptibility, it is therefore important to examine how genetic variation impacts on immunity in natural populations. So far, studies into the genetic basis of pathogen resistance in the wild have focussed almost exclusively on genes of the Major Histocompatibility Complex (MHC). The MHC is undoubtedly important in immunity to infection, but there are many other genes involved in the immune response that are yet to be investigated. Here we examine genetic variation in cytokines, signalling molecules crucial in the induction and regulation of the different effector arms of the immune response. We use a natural population of field voles, wild rodents related to common laboratory species, and show that variation within cytokine genes is linked to differences between individuals in their immune response and in resistance to multiple pathogens. Cytokines are then likely to be an important source of genetic variation to help individuals combat infection and survive in the wild.

## Introduction

Individuals vary in their resistance to infectious disease. Much of this variation is genetic and, in natural populations, considerable attention has focussed on the potential for pathogens to act as a selective force on genetic diversity [1], [2]. However, studies into the role of host genetics in infectious disease resistance in natural populations have thus far concentrated almost exclusively on genes of the Major Histocompatibility Complex (MHC) which, although important, account for only a fraction of the genetic variation in pathogen resistance [3], [4]. By contrast, a large body of literature now identifies numerous loci associated with resistance to specific pathogens in humans and domestic livestock [5]–[8]. This has obvious applied importance in establishing the genetic basis of variation in pathogen susceptibility, identifying individuals most vulnerable to infection and in determining novel molecular mechanisms of resistance. However, humans and domesticated animals inhabit environments that are divorced from the ecological context in which their immune system has evolved, typically lacking, for example, the nutritional and environmental stresses characteristic of natural populations [9]. In addition, studies of humans or domesticated animals have typically focussed on resistance to a single pathogen, whereas individuals in natural populations will be subject to concurrent or sequential infection by multiple pathogen species [10]. Thus, the selective forces and mechanisms that drive the genetic diversity involved in pathogen resistance are best studied in the context of the natural environment. More widely, differences between individuals in their ability to resist infection may have important consequences for their fitness, either in terms of survival or the ability to attract a mate and to reproduce. As such, discovering genes associated with pathogen resistance in natural populations has the potential to enable the identification of at-risk individuals or groups, to uncover novel mechanisms associated with infectious disease susceptibility or pathogenesis, and to help uncover the genetic basis of sexual selection in natural populations [4], [11].

In this paper we concentrate primarily on genes encoding cytokines. Cytokines are secreted signalling molecules that facilitate communication between cells of the immune system during both innate and acquired immune responses [12]. They are critical in the polarization, amplification and modulation of immune responses and help to determine which effector mechanisms are employed, and to what extent, in response to a given immune challenge [13]. Numerous studies in humans and domesticated animals have associated genetic variants at cytokine loci with susceptibility to specific pathogens or with host immunopathology [14]–[19]. These associations arise because different cytokine alleles vary in their immune activity or responsiveness, with consequent downstream effects on the development of immune effector functions [17], [20]. In natural populations too, genetic variation at cytokine loci could therefore underlie important differences between individuals in their immune responses or resistance to pathogens. For example, the balance between cytokines promoting Th1- and Th2-type immune responses (which are broadly effective against microparasites and macroparasites, respectively) and, in turn, the modulation of these responses by regulatory T cells, is a major factor in the outcome of the host response to infection [21], [22]. Any genetic variants that influence this balance and the resulting immune profile of an individual are likely therefore to be a major factor in determining host susceptibility [23], [24]. As different arms of the immune response are counter-regulated, and different types of pathogen require different effector responses [25], cytokine variation may be particularly relevant in natural populations infected by multiple pathogen species. Thus, one cytokine genotype may upregulate a set of immune responses effective against one class of pathogen, but this may lead to downregulation of responses that are effective against others.

To our knowledge, only two previous studies have examined how genetic variation at cytokine loci impacts on pathogen resistance in wild populations [26], [27]. Both were on ungulates (free-living Soay sheep and African buffalo) and each associated microsatellite polymorphism within a single cytokine gene (*Interferon gamma*) with resistance to gastrointestinal nematodes. Here we test, in a natural population of field voles (*Microtus agrestis*), whether coding-region polymorphisms within a wide range of cytokine and other putative resistance genes are associated with, first, differential expression of immunological parameters associated with distinct components of the immune response and, second, resistance to infection for multiple pathogen species. We use a population of wild, individually monitored field voles as a study system, where we have previously demonstrated strong interactions among the multiple pathogens infecting this host [10] and for which we have developed assays to quantify activity of distinct immunological components [28]. We find repeated associations between genetic variants at three cytokine loci with both differential immunological activity and resistance to multiple pathogens, the effects of which are of comparable magnitude to other intrinsic factors more usually considered in studies of infectious disease resistance, such as sex and body weight.

## Results

Gene fragments from 12 field vole immune genes were sequenced, yielding 26 single nucleotide polymorphisms (SNPs) from a total of 6,629 bp good quality coding sequence data (Table 1). Eighteen SNPs from eight of these genes were successfully genotyped in a total of 665 field voles. Several preliminary analyses were performed to check for any errors in the genotyping data, including testing for departures from Hardy-Weinberg equilibrium, the use of positive and negative genotyping controls and checking for unusual patterns of missing data. No unusual patterns were observed and all replicate genotype samples (*n* = 395) were successfully typed in duplicate. In addition, no genotypes were called from any of the negative controls. Finally, all animals that were genotyped at a sham *Il10* SNP were correctly called as A∶A homozygotes.

**Table 1:** Summary of sequenced field vole immune genes.*

Gene name	Brief description	2*n* a	Length^b^	SNPs discovered	SNPs genotyped	GenBank^c^
**Cytokine**						
*Interferon gamma* (*Ifng*)	Upregulation of Th1 response	24	220	0	0	HM245332
*Interleukin 1*, *beta* (*Il1b*)	Pleiotropic; pro-inflammatory	24	695	3	3	HM245333
*Interleukin 2* (*Il2*)	T cell growth factor	20	349	2	2	HM245334
*Interleukin 5* (*Il5*)	Upregulation of Th2 response	20	242	0	0	HM245335
*Interleukin 10* (*Il10*)	Anti-inflammatory	24	220	0	0	HM245336
*Interleukin 12, beta* (*Il12b*)	Larger subunit (p40) of IL-12 and IL-23; pro-inflammatory	18	520	4	2	HM245337
*Interleukin 18* (*Il18*)	Pro-inflammatory	24	410	1	0	HM245338
*Transforming growth factor*, *beta 1* (*Tgfb1*)	Anti-inflammatory/regulatory	16	937	1	1	HM245340
*Tumour necrosis factor* (*Tnf*)	Pleiotropic; pro-inflammatory	22	581	2	1	HM245343
**Non-cytokine**						
*Solute carrier family 11a member 1* (*Slc11a1*)	Ion transporter in macrophage endosomes; associated with resistance to a range of infections	16	592	2	2	HM245339
*Toll-like receptor 2* (*Tlr2*)	Pattern-recognition receptor; recognizes a variety of microbial ligands	24	937	8	4	HM245341
*Toll-like receptor 4* (*Tlr4*)	Pattern-recognition receptor; primarily recognizes LPS	18	926	3	3	HM245342
		**Total**	6629	26	18	

a Number of haplotypes sequenced.

b Length of sequence (bp).

c GenBank accession numbers for consensus sequences (including SNPs, designated using IUPAC ambiguity codes).

*We were unsuccessful in our attempts to amplify and sequence the following genes; *Il1a*, *Il4*, *Il12a* and *Il13*.

Significant linkage disequilibrium (LD) was observed between most markers located within the same gene (Table S1). No significant LD was seen between the two genotyped *Il12b* SNPs, or between several pairwise comparisons involving the *Tlr2* 1383 G/A and *Tlr2* 1706 G/A SNPs with other *Tlr2* markers. However, it should be noted that, first, minor allele frequencies are low at these loci and may not therefore give reliable estimates of LD, and second, significant LD was observed between other SNPs located within the *Tlr2* gene (Table S1). No LD was detected between SNPs located in different genes.

### Immunological variation

We tested whether genetic diversity within cytokine and other immune genes was associated with variation in immunological parameters, in animals killed as part of a cross-sectional study (*n* = 307). The immunological parameters were chosen to represent different functional arms of the immune system, and were measured as normalized mRNA expression levels, relative to a reference, pooled cDNA sample, for a suite of cytokine and transcription factor genes. Further details can be found in Materials and Methods and in [28]. Given the extent of LD observed within genes, and to reduce the number of statistical tests performed, we primarily examined the effect of haplotypes followed as necessary by post hoc single SNP analyses (see Materials and Methods). We first controlled for non-genetic intrinsic and extrinsic effects on immunological parameters. A summary of non-genetic factors associated with immunological parameters can be found in Table S2 and a more detailed analysis in [28]. The additional effect of genotype on each immunological parameter was then fitted under (*i*) an additive model, where the value of an immunological parameter is linearly related to the number of copies of a haplotype present; and (*ii*) a heterozygosity model, where heterozygotes have either increased or decreased value of an immunological parameter relative to homozygotes (i.e. molecular heterosis). Such heterosis effects have been reported acting within cytokine and other complex regulatory gene networks, possibly arising from increased stability of expression afforded by heterozygotes in a variable environment [29]–[31]. Significant associations were found between immunological parameters and genetic variation at the cytokine genes *Il1b*, *Il2* and *Il12b*. Associations were limited to these genes; no immune associations were observed with genetic variation at *Tgfb1* or *Tnf*, or with the non-cytokine membrane-bound genes (*Slc11a1*, *Tlr2* and *Tlr4*).

Polymorphism within the *Il1b* locus was strongly associated with variation in its own mRNA expression levels where, under an additive model, individuals with the GAT haplotype were associated with the highest expression (Table 2). This was the only gene where variation within the locus was associated with differential expression of that same gene. It is unlikely that the polymorphisms typed within *Il1b* in this study could themselves affect transcription, as they are located in the exonic regions of the gene. Furthermore, the individual SNP that exhibited the greatest effect (*Il1b* 324 C/T; Table S3) is synonymous and therefore does not result in an amino acid change in the translated protein. It is then perhaps more likely that the causative mutation is an un-typed polymorphism linked to the genotyped SNPs, particularly as LD is strong within this gene (Table S1). In humans, *IL1B* mRNA expression has been shown to be a heritable trait and polymorphisms within the promoter region (in particular the −31 T/C SNP) are thought to be functional [17]. It is possible that the field vole *Il1b* gene contains a polymorphism of similar effect. Identifying the precise causative mutation and the molecular mechanism of altered *Il1b* expression is however beyond the scope of this study. The *Il1b* locus was also associated with 96 hour unstimulated *Gata3* expression, with heterozygotes less likely to demonstrate a measurable *Gata3* response (Table 2), but no associations with single SNPs were observed. Marginally non-significant associations were observed between *Il1b* and expression of *Ifng* (*p* = 0.070) and *Tgfb1* (*p* = 0.056) under additive models, where for both genes the *Il1b* GGT haplotype was associated with decreased mRNA transcription. IL-1β is an important, pleiotropic mediator of the innate response and a potent pro-inflammatory cytokine. It is feasible, therefore, that polymorphism within this gene should have a discernible effect on several immunological parameters with which it interacts.

**Table 2:** Genetic terms significantly associated with variation in immunological parameters.

Genetic term	Response	Model	d.f	Term^a^	Coefficient (s.e.)	*p*-value
*Il1b*	*Il1b* expression	Additive	4	GAC	0.11 (0.17)	0.008
				GAT	0.62 (0.20)	
				AAC	−0.15 (0.22)	
				GGT	−0.02 (0.76)	
	*Gata3* 96 h expression	Heterozygosity	1	Heterozygote	−1.08 (0.52)	0.040
*Il2*	*Il10* expression	Heterozygosity	1	Heterozygote	−0.42 (0.20)	0.037
						
*Il12b*	*Il1b* expression	Additive^b^	2	CC	0.47 (0.30)	0.003
				GT	1.56 (0.49)	
	*Il2* expression	Heterozygosity^c^	1	Heterozygote	−1.15 (0.37)	0.002

a Under an additive model, listed haplotypes were compared against the most common haplotype at that locus; under a heterozygosity model, values of heterozygotes were compared against homozygotes (see text).

b Also significant under a heterozygosity model (*p* = 0.04).

c Also significant under an additive model (*p* = 0.007).

Variation within the *Il2* gene was associated with *Il10* expression under a heterozygosity model, with *Il2* heterozygotes exhibiting lower levels of *Il10* expression than homozygotes (Table 2). *Il2* heterozygotes also tended to have lower *Il1b* expression, but this trend was marginally non-significant (*p* = 0.070). Unlike *Il1b*, variation within the *Il2* gene was not associated with its own transcription levels. The observed interaction between *Il2* variation and expression of *Il10* might then relate to functional consequences of heterozygosity in *Il2* other than altered transcription levels. The two cytokines are very likely to indirectly influence each other's expression and biological activity at the protein level; IL-10 is an anti-inflammatory cytokine which inhibits the production of many other cytokines, including IL-2 [32], [33]. There is then scope for a genetic influence on the relationship between these cytokines, but further experimental work would be needed to elucidate the precise mechanism of such an interaction in the field vole.

There were strong associations between the *Il12b* locus and expression of both *Il1b* and *Il2* (Table 2). The most highly significant associations were observed under an additive model for *Il1b* expression, with the commonest *Il12b* GC haplotype linked to the lowest levels of *Il1b* expression, and the rarest GT haplotype associated with the greatest increase in *Il1b* mRNA levels. Further, under a heterozygosity model for *Il2* expression, *Il12b* heterozygotes exhibited significantly decreased *Il2* transcription. Here, however, it should be noted that because CC and GT *Il12b* haplotypes are rare (0.03 and 0.07, respectively, Table S10), these haplotypes virtually always occur as heterozygotes with the major GC haplotype. As a result, the heterozygote and additive models for *Il12b* genotype cannot be reliably distinguished and exhibit similar levels of statistical support in models of both *Il1b* and *Il2* expression. Linkage disequilibrium between SNPs within *Il12b* was low, and the observed genetic effects appear to result from associations with different SNPs. The rare C variant at the *Il12b* 278 G/C SNP was associated with markedly decreased expression of *Il2*, while the minor T allele at the *Il12b* 704 C/T locus exhibited significantly lower levels of *Il1b* expression (Table S3). Both these SNPs are nonsynonymous and may therefore be the most likely of those examined to represent causal mutations. Indeed, the *Il12b* 704 C/T SNP is predicted to have a major impact on the activity of the translated protein, based on multiple alignments and biochemical and physical characteristics of the amino acid replacements [34] (Turner *et al.*, in review). Effect sizes of the observed genetic associations with immunological parameters were equal to or greater than those related to important non-genetic factors (Table 3).

**Table 3:** Comparison of effect sizes for genetic versus intrinsic terms in immunological parameters.

Genetic term	Response	Model^a^	d.f.	Coefficient	Sex	Body weight^b^	Eye lens weight^b^
*Il1b*	*Il1b* expression	Additive	4	−0.15 to 0.62	n.s.	−0.32	−0.04
	*Gata3* 96 h expression	Heterozygosity	1	−1.08	−3.73	n.s.	−0.55
*Il2*	*Il10* expression	Heterozygosity	1	−0.42	n.s.	n.s.	n.s.
*Il12b*	*Il1b* expression	Additive	2	0.47 to 1.56	n.s.	−0.32	−0.04
	*Il2* expression	Heterozygosity	1	−1.15	n.s.	n.s.	n.s.

a Under an additive model, the range of effect sizes for alleles is shown compared against the most common haplotype at that locus; under a heterozygosity model, values of heterozygotes were compared against homozygotes (see text).

b Effect size shown for comparative purposes based on the interquartile range for females within a single season (Spring 2008).

### Pathogen resistance

**Figure 1:**
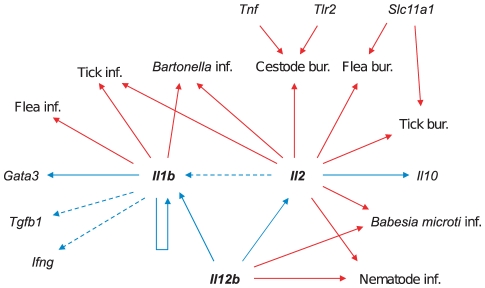
Summary diagram of genetic associations. Arrows indicate where polymorphism within a gene is associated with variation in immune parameters (blue) or pathogen resistance (red). Solid arrows represent statistically significant associations (*p*<0.05), dashed arrows represent marginally non-significant associations (0.05<*p*<0.07). ‘Inf.’ refers to probability of infection and ‘bur.’ to parasite burden. *Il1b*, *Il2* and *Il12b* were consistently associated with variation in both immune parameters and resistance to multiple pathogens.

We hypothesized that genetic variation at cytokines affecting immunological parameters would also affect pathogen resistance. We therefore tested for associations between genotypic variation and pathogen resistance in, first, the cross-sectional study in which immunological parameters were measured (*n* = 307), and second, a longitudinal study from the same study area (*n* = 358). Again, we first controlled for confounding non-genetic factors that may influence pathogen resistance (summarized in Tables S4, S5, S6, S7). We then tested for genetic associations as described for immunological parameters, above. We found that genetic variation at the cytokines *Il1b*, *Il2* and *Il12b*, which showed associations with immunological parameters, were also associated with extensive variation in resistance to a range of pathogens (Table 4). Although some other genes (*Tnf*, *Slc11a1* and *Tlr2*) also exhibited associations with certain pathogen species (Figure 1), the majority of associations were observed for the cytokines *Il1b*, *Il2* and *Il12b* and, in each case, against multiple pathogen species. As these were also the only genes associated with variation in immunological parameters, we will therefore concentrate primarily on these.

**Table 4:** Genetic terms significantly associated with variation in pathogen resistance.

Genetic term	Response	Dataset a	Model	d.f.	Term^b^	Coefficient (s.e.)	*p*-value	ΔAIC c
*Il1b*	Tick infection	CS	Heterozygosity	1	Heterozygote	0.91 (0.41)	0.021	-
	*Bartonella* infection	Long.	Heterozygosity	1	Heterozygote	−0.61 (0.23)	0.008*	4.6
	Flea infection	Long.	Additive^d^	4	GGC	0.36 (0.19)	0.053*	2.2
					GAT	−0.22 (0.21)		
					AAC	−0.25 (0.25)		
					GGT	1.27 (1.23)		
*Il2*	Nematode infection	CS	Additive^e^	2	AG	−0.03 (0.26)	0.043	-
					TC	0.70 (0.29)		
	Cestode burden	CS	Heterozygosity	1	Heterozygote	−0.45 (0.14)	0.001	-
	Flea burden	CS	Heterozygosity	1	Heterozygote	−0.27 (0.14)	0.049	-
	Tick infection	Long.	Additive	2	AG	0.21 (0.19)	0.122*	2.3
					TC	−0.66 (0.34)		
	Tick burden	CS	Heterozygosity	1	Heterozygote	0.58 (0.28)	0.038	-
	*Babesia* infection	Long.	Heterozygosity	1	Heterozygote	0.66 (0.23)	0.002*	7.2
	*Bartonella* infection	Long.	Heterozygosity	1	Heterozygote	−0.61 (0.21)	0.004*	5.9
*Il12b*	Nematode infection	CS	Additive^f^	2	CC	1.42 (0.44)	0.006	-
					GT	0.42 (0.75)		
	*Babesia* infection	Long.	Additive	2	CC	1.02 (0.31)	0.003*	6.8
					GT	0.26 (0.77)		
*Slc11a1*	Flea burden	CS	Heterozygosity	1	Heterozygote	−0.28 (0.14)	0.046	-
	Tick burden	CS	Heterozygosity	1	Heterozygote	0.67 (0.28)	0.015	-
*Tlr2*	Cestode burden	CS	Heterozygosity	1	Heterozygote	0.37 (0.14)	0.007	-
*Tnf*	Cestode infection	CS	Additive	1	T	−0.68 (0.22)	0.001	-

a Refers to cross-sectional (CS) or longitudinal (Long.) studies, which utilized GLMs and GLMMS, respectively, for analyses (see text).

b Under an additive model, listed haplotypes were compared against the most common haplotype at that locus; under a heterozygosity model, values of heterozygotes were compared against homozygotes (see text).

c Increment in AIC of the model if single term is dropped (GLMMs only).

d Also significant under a heterozygosity model (ΔAIC = 2.1).

e Also significant under a heterozygosity model (*p* = 0.043).

f Also significant under a heterozygosity model (*p* = 0.007).

*P-values given for analyses using GLMMs are for equivalent GLMs.

*Il1b* haplotype was associated with variation in the probability of infestation by ticks and fleas and infection with *Bartonella* (Table 4). *Il1b*-heterozygous individuals were more likely to be infested with ticks and less likely to be infected with *Bartonella* or infested with fleas. Furthermore, under an additive model, voles that carried the GGC or GGT haplotypes were more likely to be infested with fleas. Post hoc single SNP analyses revealed that the association with the probability of flea infestation was largely attributable to the nonsynonymous *Il1b* 253 A/G SNP, with individuals carrying the G allele at this locus more likely to be infested (Table S8).

Polymorphism within the *Il2* gene was associated with resistance to a wide range of pathogens (Table 4). Significant associations under additive models were observed between *Il2* and infection by nematodes and tick infestation; the TC haplotype was associated with an increased chance of infection by nematodes but, in the longitudinal study, a decreased probability of tick infestation. Voles heterozygous at the *Il2* locus were more likely to harbour a *Babesia microti* infection but less likely to be infected with *Bartonella*. Furthermore, in the cross sectional study, heterozygosity at the *Il2* locus was associated with lower numbers of cestodes and fleas, but an increased tick burden (Table 4).

Variation within the vole *Il12b* gene was associated with resistance to nematodes and to *Babesia microti*, each under an additive model (Table 4). In both cases, individuals carrying the *Il12b* CC haplotype had an increased probability of infection. Single SNP analyses revealed that these associations were due solely to the *Il12b* 273 G/C SNP, where the C allele increased probability of infection; no effect of the *Il12b* 704 T/C SNP was observed (Table S8). In all cases, the effect sizes found for cytokine variants were of comparable magnitude to those found for other intrinsic, but non-genetic, factors such as sex and body weight (Table 5). Only season, an extrinsic factor, consistently showed stronger effects on probability of pathogen infection. Although no previous studies have examined associations between *Il1b*, *Il2*, or *Il12b* and infectious disease in a wild species, their roles in the shaping of immune responses means it is not difficult to imagine that polymorphism within these genes may affect pathogen resistance, particularly given the associations with immunological parameters described above.

**Table 5:** Comparison of effect sizes for genetic versus intrinsic terms in pathogen resistance.

Genetic term	Response	Dataset a	Model^b^	d.f.	Coefficient	Sex	Body weight^c^	Eye lens weight^c^
*Il1b*	Tick infection	CS	Heterozygosity	1	0.91	0.35	1.11	−0.74
	*Bartonella* infection	Long.	Heterozygosity	1	−0.61	n.s.	n.s.	0.32
	Flea infection	Long.	Additive	4	−0.25 to 1.27	0.72	−0.04	n.d.
*Il2*	Nematode infection	CS	Additive	2	−0.03 to 0.70	0.82	n.s.	n.s.
	Cestode burden	CS	Heterozygosity	1	−0.45	−1.61	0.16	−0.33
	Flea burden	CS	Heterozygosity	1	−0.27	n.s.	0.53	n.s.
	Tick infection	Long.	Additive	2	−0.66 to 0.21	n.s.	0.15	n.d.
	Tick burden	CS	Heterozygosity	1	0.58	n.s.	2.68	n.d.
	*Babesia* infection	Long.	Heterozygosity	1	0.66	−0.70	0.32	n.d.
	*Bartonella* infection	Long.	Heterozygosity	1	−0.61	−0.22	−0.47	n.d.
*Il12b*	Nematode infection	CS	Additive	2	0.42 to 1.42	0.82	n.s.	n.s.
	*Babesia* infection	Long.	Additive	2	0.26 to 1.02	−0.70	0.32	n.d.
*Slc11a1*	Flea burden	CS	Heterozygosity	1	−0.28	n.s.	0.53	n.s.
	Tick burden	CS	Heterozygosity	1	0.67	n.s.	2.68	n.d.
*Tlr2*	Cestode burden	CS	Heterozygosity	1	0.37	−1.61	0.16	−0.33
*Tnf*	Cestode infection	CS	Additive	1	−0.68	0.88	0.46	−0.70

a Refers to cross-sectional (CS) or longitudinal (Long.) studies, which utilized GLMs and GLMMS, respectively, for analyses (see text).

b Under an additive model, the range of effect sizes for alleles is shown compared against the most common haplotype at that locus; under a heterozygosity model, values of heterozygotes were compared against homozygotes (see text).

c Effect size shown for comparative purposes based on the interquartile range for females within a single season (Spring 2008).

## Discussion

Twin aims of this study were, first, to expand research on wildlife immunogenetics away from concentrating solely on the MHC, and second, to transfer the broader research conducted on laboratory species into the more ecologically valid arena of natural populations. We have previously demonstrated strong interactions between the multiple pathogens infecting the Kielder voles [10] and associations between seasonal and non-genetic host factors and individual variation in immunological parameters [28]. In the present study, we have explicitly controlled for such confounding non-genetic factors and have demonstrated that genetic variation at cytokine genes provides a source for phenotypic variation in both immunological parameters and in pathogen resistance in the natural environment. We have concentrated primarily on cytokines as these genes play critical roles in many physiological and immunological processes, yet, despite being well studied in humans and model organisms, have thus far been overlooked in studies of disease resistance in natural populations.

The analyses of this paper necessarily required a relatively large number of statistical tests of association, yet explicitly correcting for this (by Bonferroni methods) would likely be too conservative and could lead to the rejection of truly significant genetic associations. However, non-independence among tests – inherent in any study of a cytokine network – prevents the assignment of accurate false discovery rates. Instead, we emphasize in this discussion only those genes that exhibited repeated associations with both immunological parameters and pathogen resistance, since while single associations are inevitably prone to Type I Errors, these are unlikely where associations are repeated and consistent.

Polymorphisms within three cytokine genes, *Il1b*, *Il2* and *Il12b*, were associated with variation in important components of the immune response in our natural populations of field voles (Figure 1). Effects were localized to these genes; no associations with immunological parameters were observed either for other cytokines or for any of the membrane-bound genes. Furthermore, variation within these three genes were also associated with resistance to multiple pathogen species which, taken together, is consistent with effects of genetic variation in pathogen resistance being mediated by variation in systemic immune responsiveness between cytokine genotypes. IL-1β, IL-2 and IL-12 are all broadly immunostimulatory cytokines which, through acting on the same cells, interact indirectly with one another during both the innate and adaptive immune response [16], [32]. In this study, these three genes exhibited associations with each other (Table 2; Figure 1), which is suggestive of a possible shared physiological pathway in which interactions may occur within the field vole. The IL-12 p40 subunit encoded by *Il12b* also forms part of the related cytokine IL-23, which is another mediator of the inflammatory response and is involved in the production of Th17 cells [35], [36]. There are then a number of possible pathways in which *Il1b*, *Il2* and *Il12b* may interact *in vivo*, giving rise to the observed associations. Elucidating the precise mechanisms and pathways underlying such immunological interactions in wild rodents represents an exciting avenue for future work. The fact that associations are localized to these three cytokines also argues against these associations having arisen spuriously due to demographic factors, such as population subdivision or inbreeding, since any such demographic factors would act equally at all loci [37], not just *Il1b*, *Il2* and *Il12b*. Furthermore, any systematic differences between sites were tested and controlled for in our statistical models. Finally, in a separate study, we show that there is no evidence for subdivision or inbreeding in the populations studied, based on the markers presented here [34] (Turner *et al.*, in review).

The magnitude of the effects observed for genetic variation at *Il1b*, *Il2* and *Il12b* on immunological parameters and pathogen resistance were of comparable size to those observed for other important intrinsic factors, such as sex and weight, which are more usually considered in studies of infectious disease susceptibility. Cytokine polymorphism therefore represents an important source of variation between individuals in immune function and pathogen resistance. This variation may, in part, be maintained by exposure to multiple pathogen species, such that a genotype conferring resistance to one pathogen species may confer increased susceptibility to a second (e.g. *Il2* TC haplotypes are associated with lower probability of tick infestation but an increased risk of nematode infection, Table 4). These associations may represent an example of antagonistic pleiotropy, which has been proposed as a mechanism by which genetic diversity may be maintained within a population [38], [39]. In support of this, we have shown elsewhere that high sequence and allelic diversity observed within field vole *Il1b* and *Il2* genes appears to have been maintained through natural selection [34] (Turner *et al.*, in review). Alongside their direct immunological roles, cytokines also have a wide-range of non-immune physiological effects including influences on thermoregulation, appetite and fatigue [40]. It is therefore likely that an alteration in the action of cytokines will have a more significant effect on host pathology than simply its impact on infectious disease susceptibility [41]. Natural selection for resistance to a pathogen may lead to an increase in frequency of alleles that are detrimental in the absence of infection [42]. These antagonistic effects on fitness may be particularly likely to occur for immune gene polymorphisms, as many immune responses to infection, such as inflammation, can actually be harmful to host tissues. It is known in humans that the magnitude of cytokine-mediated inflammatory responses has a genetic basis and, while an effective immune response is essential for the clearance of pathogenic organisms, too strong a response can lead to significant immunopathology [43]. It is notable in this respect that *Il2* polymorphism was associated both with resistance to a wide range of pathogens and to *Il10* expression (Table 2 and Table 4), and that IL-10 is an anti-inflammatory cytokine associated with preventing immunopathology [44]. The complex nature of the often antagonistic genetic associations observed for *Il1b*, *Il2* and *Il12b* may therefore represent a balance between normal physiological functioning and an ability to deal effectively with an unpredictable and dynamic range of co-infecting pathogens.

Our results suggest that wildlife immunogenetics should broaden its scope beyond MHC genes [4] and that cytokine variation in natural populations may have a number of equally important consequences. First, cytokine variation may help to provide populations with resilience against infectious disease [41] and so is likely to be an important source of genetic variation to conserve in endangered species. Second, our results suggest that cytokines may represent a source of ‘good genes’ as predicted by sexual selection and the immunocompetence handicap hypothesis; genes that protect males against pathogens prevalent in the environment may allow them to invest more in sexually selected traits, thereby indicating their fitness to females [45], [46].

Finally, we note that by studying wild rodent populations we have the opportunity to extend laboratory immunology to the field [47]. Wild rodents exhibit increased and much more variable immune responses than their laboratory relatives [48]. To better understand variation in susceptibility to infection – including genetic components of this variation – we must therefore examine immune responses within the ecological context in which they have evolved. In addition, because of their relatedness to traditional laboratory species, the transfer of laboratory-based immunology and genetics to wild rodents should be relatively straightforward. We also highlight the importance of measuring multiple immune parameters in natural populations to move beyond the simplified view of ‘immunocompetence’ as a single trait [12], [49]. Thus, while we do not underestimate the importance of simple measures of immunity (such as phytohemagglutinin-induced swelling) which have thus far predominated in ecological studies, a one-off measure of a single aspect of immunity is unlikely to give an accurate representation of an individual's ‘immunocompetence’ [50]. The ability to monitor the various arms of the immune response (innate, adaptive, regulatory etc.), and account for individual variation, will help us to better define the immune status of hosts and may therefore increase our understanding of how host immune profile influences life-history traits, co-infection dynamics, susceptibility to pathogens and disease transmission [12], [25], [28]. As cytokine function tends to be conserved across taxa, incorporating measurements of these important molecules into studies of ecological immunology will offer insights into many different host-parasite interactions [49]. Wild rodents may therefore provide a new model in ecological immunology, with great potential to begin to bridge the gap in our understanding between the mechanistic insights of immunity gained through studies on laboratory rodents and the variation in infectious disease susceptibility and immune responsiveness observed in humans and other natural populations.

## Materials and Methods

### Ethics statement

All animal procedures were performed under UK Home Office licence (40/3235) and with approval from the University of Liverpool Animal Welfare Committee.

### Study site and animals

Naturally-occurring field voles were sampled, first, by a cross-sectional study involving destructive sampling of field voles followed by both immunological and pathogen assays (*n* = 307) [28] and, second, by a longitudinal capture-recapture study accompanied by pathogen assays (*n* = 358). Briefly, two grassy clear-cut sites within Kielder Forest, UK, designated SQC (55.2549, −2.6116) and BLB (55.2457, −2.6108), were sampled monthly. For the cross-sectional study (February to November 2008 and February and March 2009), curvilinear transects of 100 Ugglan special live capture traps (Grahnab, Sweden), arranged at 5–10 m intervals, were placed around the margins of each habitat in order to sample a very large area of the habitat and provide data representative of the whole clear-cut population. For the longitudinal study (March to October 2008), a rectangular 0.375 ha, 150-trap (10×15) live-trapping grid was placed centrally in each habitat, and individual voles were tagged by injecting a subcutaneous Passive Induced Transponder (PIT) tag (AVID plc., Uckfield, UK) under the skin at the back of the neck, and subsequently identified via their unique nine-digit code with hand-held scanners (AVID). In November 2008 and March 2009, larger samples of animals were captured and killed from both transect and central grid habitats at both sites, including animals that had been previously marked with transponders and processed as part of the capture-recapture study. On capture, each animal was weighed, sexed and examined for ectoparasites (see ‘Pathogen assays’, below). Reproductive maturity was also ascertained; males were classed as sexually mature if they possessed an adult coat and showed external signs of descended testes, while females were designated as mature if they had an adult coat along with a perforate vagina and/or an open pubic symphysis and evidence of lactation. Animals caught as part of the longitudinal study were sampled non-invasively by acquiring a small amount of blood from the tip of the tail, which was placed immediately in RNA*later* (Qiagen, Crawley, UK) and stored at −80°C as soon as possible.

### SNP discovery and genotyping

We chose an initial panel of 16 candidate genes, 13 cytokines and 3 non-cytokines (Table 1). The identification of single nucleotide polymorphisms (SNPs) within these genes is described fully elsewhere [34] (Turner *et al.*, in review). Briefly, degenerate primers were designed from alignments of rat and mouse coding sequence and used to amplify cDNA from approximately 12 individual field vole spleens. We were able to amplify and sequence 12 of our original set of 16 candidate genes, designing genomic assays for eight of these. These included the following five cytokine genes; *Interleukin 1*, *beta* (*Il1b*), *Il2*, *Il12b*, *Transforming growth factor*, *beta 1* (*Tgfb1*) and *Tumour necrosis factor* (*Tnf*). SNPs from three non-cytokine genes were also analysed; *Solute carrier family 11a*, *member 1* (*Slc11a1*), *Toll-like receptor 2* (*Tlr2*) and *Tlr4*. These genes have previously been implicated in pathogen resistance but, unlike cytokines, are all membrane bound and so lack systemic activity. No SNPs were identified in *Ifng*, *Il5* or *Il10*, and we could not successfully genotype the single *Il18* SNP; these genes were therefore excluded from this study. 9 lists the primers and PCR conditions used to amplify the gene products above. We were unable to amplify the field vole cytokine genes *Il1a*, *Il4*, *Il12a* and *Il13* despite several attempts to redesign primers and optimize PCR conditions.

Genomic DNA was extracted from the livers of voles caught and killed as part of the cross-sectional study (*n* = 307), using DNeasy Blood and Tissue Kit (Qiagen). For the non-invasive longitudinal samples (*n* = 358), gDNA was extracted from tail blood using DNAzol BD Reagent (Invitrogen, Paisley, UK), specifically using the protocol of Mackey *et al.* [51], which is modified for extracting DNA from small volumes of blood; the resulting gDNA pellet was resuspended in 12.5 µl water and 1 µL of this solution was used as a template for whole genome amplification, using the illustra GenomiPhi V2 DNA amplification kit (GE Healthcare, Buckinghamshire, UK). Genotyping was performed by KBioscience (Hoddesdon, UK; http://www.kbioscience.co.uk) utilizing the KASPar SNP genotyping system, their own novel form of competitive allele-specific PCR, and incorporated duplicate samples, negative controls (water and blank whole genome amplification reactions) and a positive control (a monomorphic site in *Il10* to check for miscalling rates) to validate reproducibility.

### Preliminary analyses

Several preliminary analyses were performed as checks on the SNP genotyping data, including examination of controls and for unusual patterns of missing data. Deviations from Hardy-Weinberg equilibrium were tested for in GENEPOP 4.0 [52]–[54], using the Markov chain method to evaluate exact tests. The extent of linkage disequilibrium (LD) between SNPs was analysed using LinkDos [55], which computes genotypic or composite linkage disequilibrium estimates from diploid data [56]–[58]. Because of the large number of LD tests performed, a sequential Bonferroni correction [59], [60] was applied to the resulting *p*-values. Pairwise LD measurements were calculated for every pair of SNPs, to test the hypotheses that SNPs within the same gene would demonstrate high LD – and thus have greater power to pick up a phenotypic association if a causal mutation was up or downstream from the genotyped polymorphism – and that SNPs located in different genes would not demonstrate LD and genetic loci were therefore independent.

Haplotypes were inferred from genotypic data using algorithms provided by PHASE v2.1 [61], [62] within DnaSP v5 [63], using the default settings of 100 main iterations, a thinning interval of 1 and 100 burn-in iterations. To check reliability of the haplotype reconstruction, the algorithm was run five times for each data set and checked for consistency across the runs; no modifications of the default settings were necessary. PHASE imputes haplotypes even when genotypic data for an individual is missing; however, if more than one SNP per gene was missing for a particular individual the resulting inferred haplotype was deemed unreliable and excluded from further analyses. A summary of haplotype frequencies for each gene can be found in Table S10.

### Immunological assays

Relative mRNA expression of several cytokines and immunity-related transcription factors was measured using quantitative real-time PCR (Q-PCR), from cultured leucocytes isolated from spleens of field voles killed as part of the cross sectional study (*n* = 307) [28]. Messenger RNA accumulations were measured from both unstimulated cells, reflecting constitutive levels of mRNA expression, and from cells cultured with various immunostimulatory molecules, under the assumption that observed *ex vivo* responses reflect the potential for that response to occur *in vivo* [28]. We note that protein-based assays, such as ELISAs, were not available for this species.

A summary of the immunological assays can be found in Table S11. mRNA accumulations of the following genes were measured at 24 hours: *Il1b*, *Interferon regulatory factor 5* (*Irf5*; a transcription factor which regulates the production of type I interferons and inflammatory cytokines [64]), *Tgfb1* and *Il10*, for both constitutive expression and for splenocyte cultures stimulated with either the TLR2 agonist HKLM (heat-killed *Listeria monocytogenes*) or the synthetic TLR7 agonist, imiquimod (InvivoGen, San Diego, USA). The production of *Irf5* mRNA in the TLR2-stimulated culture and *Il1b* in TLR7-stimulated cells did not demonstrate a positive response to the stimuli after analysis of 100 individuals and so these measurements were ceased [28].

mRNA expression was measured at 96 hours for *Ifng*, *Il2*, *T-box21* (*Tbx21/Tbet*; a transcription factor which is specific to the Th1 cell subset [65]), *Gata binding protein 3* (*Gata3*; a transcription factor associated with the Th2 cell subset [66]), *Forkhead box p3* (*Foxp3*; another transcription factor, associated with regulatory T cells [67]), *Tgfb1* and *Il10*. Expression levels were measured in both unstimulated splenocyte cultures and cultures stimulated with the mitogen phytohaemagglutinin (PHA), which preferentially induces proliferation of activated CD4^+^ helper T cells [68]. For all assays, target genes were first normalized to the endogenous control gene *Ywhaz* (tyrosine 3-monooxygenase/tryptophan 5-monooxygenase activation protein, zeta polypeptide) and then measured and analysed as normalized expression relative to a reference, pooled cDNA sample (the ΔΔ Ct method [69]). This method allows one to compare relative, normalized gene expression levels between animals [28].

### Pathogen assays

On capture, all animals were thoroughly examined for ticks and fleas. Counts of three species of flea (*Peromyscopsylla spectabilis*, *Ctenophthalmus nobilis vulgaris*, *Megabothris walkeri*) were grouped as a single ‘flea’ variable in order to simplify further analyses, under the assumption that they have a similar effect on the host. Field voles caught as part of the cross-sectional study were killed by an overdose of chloroform followed by exsanguination. Each animal was then dissected and examined for infection by nematodes and cestodes. Total abundances were measured and analysed for several species; however, in order to simplify analyses and improve sample size, data were reduced and recorded as presence or absence of cestodes or nematodes. In addition, overall burdens of all cestodes were analysed for each infected vole. Animals caught as part of the longitudinal study were sampled non-destructively via tail bleeds and could not therefore be examined for helminths, with the exception of those animals captured and killed in November 2008 and March 2009.

Field voles from both studies were tested for infection by two blood-borne pathogens using specific PCR protocols described previously [70], [71]: *Babesia microti*, a tick-transmitted protozoan parasite, and *Bartonella* spp., which are flea-borne, gram-negative bacteria. Although up to five species of *Bartonella* circulate concurrently in rodent communities in the UK [72]–[74], all species are thought to have similar life-cycles and are predicted to interact with the host immune system in a similar way [75]; therefore, individual voles were recorded as either positive or negative for the presence of *Bartonella* spp.

### Statistical analysis

Statistical modelling was performed using R version 2.10 [76]. The primary aim of the analyses was to examine the relationship between genetic variation and both immune function and pathogen resistance, whilst controlling for confounding non-genetic factors. The approach, for each response variable, was first to construct a minimal model containing only non-genetic terms. To this, each genetic term was added in turn to assess their significance in explaining the remaining variance for a given response variable. We chose to primarily examine genetic associations using haplotypes rather than single SNPs, for several biological and statistical reasons. First, the functional and biologically relevant product of a gene, the protein, consists of chains of amino acids whose sequences correspond to haplotypes inherited from each parent [77]. Second, variation in populations is structured into haplotype blocks which are likely to be transmitted as a single unit [77], [78]. Third, utilizing haplotypes comprised of multiple SNPs reduces the number of tests performed in comparison to testing each individual SNP. Finally, it has been recognized that haplotype-based studies are more powerful for detecting phenotypic associations resulting from untyped causal mutations in LD with genotyped SNPs/haplotypes [78], [79]. However, in addition to haplotype-based analyses, post hoc single SNP analyses were also carried out where appropriate in an attempt to identify any potential causal mutations.

Host immunological parameters were measured via the quantification of expression levels of a number of immune genes, with multiple, positively correlated measurements taken of mRNA expression of each gene (for example, innate, unstimulated expression and TLR-stimulated expression levels). Variables from the same gene were therefore grouped together, as follows. All variables were first log+1 transformed and then standardized by subtracting the mean and dividing by the standard deviation. Where two positively correlated measurements were made on the same gene, these were summed. Where more than two positively correlated measures were made, a principal components analysis (PCA) was carried out and the first principal component extracted [28]. By combining correlated measurements into a single variable for each gene, we aimed to reduce the number of statistical tests performed and also simplify interpretation by providing single variables incorporating aspects of both constitutive and potential maximal expression. In addition, as grouped measurements are likely to dilute the effect of any given single measurement, this should lead to stronger inference and more robust genetic associations. *Gata3* expression values could not be combined as the two quantitative measurements taken were poorly correlated. Therefore, each *Gata3* response variable was analysed separately. A summary of the immune response variables derived from standardized scores or PCA can be found in Table S12. For each of these response variables, linear models were used to construct non-genetic models containing the following terms as main effects along with their two way interactions: site (two levels: SQC and BLB); sex (2 levels: male and female); season [five levels, designated as: spring 2008 (March to May 2008), summer 2008 (June to August 2008), autumn 2008 (September to November 2008), winter 2008 (December 2008 to February 2009) and spring 2009 (March 2009)]; body weight (continuous) and eye-lens weight (continuous). Model simplification was then performed by deletion testing, to remove non-significant terms and create a base (‘minimal’) non-genetic model. Non-genetic models for *Gata3* were constructed as above, except that a Box-Cox transformation [80] was performed on *Gata3* mitogen-stimulated data, and a generalized linear model (GLM) with a binomial error distribution was performed on *Gata3* unstimulated (detectable response/no detectable response) data. Haplotypes at each locus were tested for association with each response variable by fitting these genetic terms to the minimal non-genetic models as either (*i*) an additive model, where the effect, *α_i_*, of a haplotype *A_i_* relative to haplotype *A_0_* is assumed to be 0, *α_i_* and 2*α_i_* for genotypes *A_0_A_0_*, *A_0_A_i_* and *A_i_A_i_*, respectively, or (*ii*) a heterozygosity model, where, for each gene, values of heterozygotes were compared to homozygotes. In some instances, both additive and heterozygote models returned significant results for the same locus, in which case the *p*-values for both terms are reported but the model with the lowest *p*-value is presented fully.

Pathogen variables from the cross-sectional data were analysed by GLMs using either a binomial error structure with a logit-link function (for binary, presence/absence variables) or quasi-Poisson errors with a log-link function (for count data which exhibited overdispersion). Non-genetic models were constructed as above. For the longitudinal data, pathogen variables were analysed using generalized linear mixed models (GLMMs) with a binomial error distribution, and fitted using restricted estimate maximum likelihood (REML) by the lmer function in R, for the following variables: site; sex; season [spring 2008 (March to May 2008), summer 2008 (June to August 2008), autumn 2008 (September to November 2008), winter 2008 (December 2008 to February 2009)]; maturity status (two levels, immature and currently/previously reproductively active); body weight (standardized by subtraction of the mean weight from each individual value); and recapture status (whether or not the animal had been caught previously). Two-way interactions were considered for all terms except recapture status. As animals caught at the same site in the same trap session experienced the same environmental conditions, site*trap session was included as a random effect [81]. To account for the correlation amongst different observations of the same individual, *individual* was also added as a random effect to the non-genetic model. However, due to problems with model convergence, *individual* could not be added to the model of *Babesia microti* infection. The Akaike Information Criterion (AIC) index [82] was used to select the most parsimonious non-genetic minimal model such that terms were discarded sequentially until no term could be removed without causing an increase in AIC of greater than two [83]. Genetic terms were tested under heterozygote and additive models, with significance assessed using either likelihood ratio tests (GLMs) or AIC (GLMMs). Additionally, for GLMMs, we also constructed equivalent GLMs as a check for consistency (i.e. to check that a significant association observed in a mixed model was also observed in a GLM) and to ensure that the use of mixed models and addition of random effects had not led to problems with model convergence.

Infection data were left out of the non-genetic models for the sake of parsimony. However, current infection status is likely to impact both on host immunological parameters and resistance to other pathogen species. We therefore performed post hoc tests to examine whether observed genetic associations remained after addition of pathogen data as explanatory variables. The addition of infection status had no effect on the significance of genetic associations with either immunological parameters or resistance to other pathogens.

## Supporting Information

Table S1 Pairwise linkage disequilibria between pairs of SNPs located within the same gene.
(DOC)

Table S2 Linear models describing non-genetic factors associated with variation in immune gene expression.
(DOC)

Table S3 Post-hoc single SNP associations with immune profile.
(DOC)

Table S4 GLMs describing non-genetic factors associated with the probability of pathogen infection.
(DOC)

Table S5 GLMs describing non-genetic factors associated with variation in pathogen burden.
(DOC)

Table S6 GLMMs describing non-genetic factors associated with variation in the probability of pathogen infection.
(DOC)

Table S7 GLMMs describing non-genetic factors associated with variation in tick burden.
(DOC)

Table S8 Post-hoc single SNP associations with pathogen resistance.
(DOC)

Table S9 Primers and PCR conditions used to amplify gene products from cDNA.
(DOC)

Table S10 Haplotype frequencies inferred from genotyping data for the combined cross-sectional and longitudinal datasets.
(DOC)

Table S11 Summary of splenocyte culture assays and mRNA measurements taken.
(DOC)

Table S12 Immune response variables derived from combining multiple measurements.
(DOC)
