# Supplementary material for: Genetic Diversity in Cytokines Associated with Immune Variation and Resistance to Multiple Pathogens in a Natural Rodent Population
Source: PLoS Genet. 2011 Oct 20;7(10):e1002343. doi: 10.1371/journal.pgen.1002343 (PMC3197692; doi:10.1371/journal.pgen.1002343)
Supplement: Table S1 — Pairwise linkage disequilibria between pairs of SNPs located within the same gene. (DOC) [file pgen.1002343.s001.doc]

Table S1 Pairwise linkage disequilibria between pairs of SNPs located within the same gene. Blanks, no LD coefficient returned by LinkDos when LD is non-significant.

| **Gene** | **SNP locus 1** | **SNP locus 2** | ***Δ*_ij_^a^** | ***R*_ij_^b^** | ***P^c^*** |
| --- | --- | --- | --- | --- | --- |
| *Il1b* | *Il1b* 243 G/A | *Il1b* 253 A/G | 0.043 | 0.251 | **0.0001** |
|  | *Il1b* 243 G/A | *Il1b* 324 C/T | 0.038 | 0.255 | **0.0001** |
|  | *Il1b* 253 A/G | *Il1b* 324 C/T | 0.055 | 0.284 | **0.0001** |
| *Il2* | *Il2* 381 A/T | *Il2* 408 C/G | 0.041 | 0.262 | **0.0001** |
| *Il12b* | *Il12b* 278 G/C | *Il12b* 704 C/T | - | 0.019 | 0.650 |
| *Slc11a1* | *Slc11a1* 537 C/G | *Slc11a1* 714 G/A | 0.182 | 0.943 | **0.0001** |
| *Tlr2* | *Tlr2* 1383 G/A | *Tlr2* 1648 G/A | - | 0.058 | 0.157 |
|  | *Tlr2* 1383 G/A | *Tlr2* 1706 G/A | - | 0.014 | 0.733 |
|  | *Tlr2* 1383 G/A | *Tlr2* 1944 T/C | 0.020 | 0.181 | **0.0001** |
|  | *Tlr2* 1648 G/A | *Tlr2* 1706 G/A | - | 0.020 | 0.635 |
|  | *Tlr2* 1648 G/A | *Tlr2* 1944 T/C | 0.035 | 0.248 | **0.0001** |
|  | *Tlr2* 1706 G/A | *Tlr2* 1944 T/C | - | 0.025 | 0.546 |
| *Tlr4* | *Tlr4* 1663 A/G | *Tlr4* 1848 G/T | 0.020 | 0.585 | **0.0001** |
|  | *Tlr4* 1663 A/G | *Tlr4* 2037 C/A | 0.048 | 0.928 | **0.0001** |
|  | *Tlr4* 1848 G/T | *Tlr4* 2037 C/A | 0.019 | 0.620 | **0.0001** |

^a^ Unbiased linkage disequilibrium coefficient, defined as *Δ_ij_*  = (*N/N* – 1)((*T_ij_*/*N*) – 2*p_i_p_j_)*, where *T_ij_* is the number of times that the alleles *i* and *j* appear in the same individual, *N* is the total number of genotypes, while *p_i_* and *p_j_* indicate the frequency of the *i*^th^ allele at one locus and the *j*^th^ allele at another locus, respectively [57].

^b^ Correlation coefficient, defined as *R_ij_* = *Δ_ij_* / ((*p_i_*(1 – *p_i_*)+*C_i_*) (*p_j_*(1 – *p_j_*)+*C_j_*))^1/2^, where the *C* term is a correction for departures from random mating; *C_i_* is equal to the observed minus the expected frequencies of homozygotes for the *i*^th^ allele [57].

^c^ *P*-values ascertained from Chi-squared statistic, *χ^2^* = *NR_ij_^2^* [58].
