# Supplementary material for: Genetic Diversity in Cytokines Associated with Immune Variation and Resistance to Multiple Pathogens in a Natural Rodent Population
Source: PLoS Genet. 2011 Oct 20;7(10):e1002343. doi: 10.1371/journal.pgen.1002343 (PMC3197692; doi:10.1371/journal.pgen.1002343)
Supplement: Table S2 — Linear models describing non-genetic factors associated with variation in immune gene expression. (DOC) [file pgen.1002343.s002.doc]

Table S2 Linear models describing non-genetic factors associated with variation in immune gene expression.

| **Term** | **Coefficient** | **s.e** | ***t*-value** | ***p*-value** |
| --- | --- | --- | --- | --- |
| *Foxp3* expression |  |  |  |  |
| Intercept | -1.27 | 0.98 | -1.29 | 0.198 |
| Site (SQC) | -0.95 | 0.89 | -1.07 | 0.286 |
| Season (summer 2008) | 1.01 | 0.46 | 2.21 | 0.025 |
| Season (autumn 2008) | 1.71 | 0.49 | 3.46 | <0.001 |
| Season (winter 2008) | 1.12 | 0.50 | 2.26 | 0.026 |
| Season (spring 2009) | 1.30 | 0.62 | 2.10 | 0.037 |
| Body weight | 0.04 | 0.03 | 1.14 | 0.258 |
| Eye lens weight | -23.36 | 221.55 | -0.10 | 0.916 |
| Sex (male) | -0.72 | 0.38 | -1.90 | 0.059 |
| Site (SQC) × body weight | -0.11 | 0.04 | -2.34 | 0.020 |
| Site (SQC) × eye lens weight | 666.55 | 262.86 | 2.53 | 0.012 |
| Site (SQC) × sex (male) | 1.18 | 0.52 | 2.25 | 0.026 |
| *Gata3*, 96 h PHA^a^ expression |  |  |  |  |
| Intercept | 0.21 | 0.07 | 3.18 | 0.002 |
| Season (summer 2008) | -0.17 | 0.10 | -1.80 | 0.074 |
| Season (autumn 2008) | 0.16 | 0.11 | 1.49 | 0.136 |
| Season (winter 2008) | -0.13 | 0.13 | -1.01 | 0.316 |
| Season (spring 2009) | -0.07 | 0.18 | -0.38 | 0.706 |
| Body weight | -0.003 | 0.002 | -1.23 | 0.220 |
| Season (summer 2008) × body weight | 0.007 | 0.003 | 2.27 | 0.024 |
| Season (autumn 2008) × body weight | -0.007 | 0.005 | -1.43 | 0.154 |
| Season (winter 2008) × body weight | 0.004 | 0.006 | 0.63 | 0.529 |
| Season (spring 2009) × body weight | 0.003 | 0.009 | 0.31 | 0.758 |
| *Ifng* expression |  |  |  |  |
| Intercept | -1.59 | 0.64 | -2.48 | 0.014 |
| Site (SQC) | 0.63 | 0.27 | 2.34 | 0.020 |
| Season (summer 2008) | 0.05 | 0.40 | 0.11 | 0.910 |
| Season (autumn 2008) | -0.75 | 0.39 | -1.95 | 0.053 |
| Season (winter 2008) | 1.70 | 0.40 | 4.27 | <0.001 |
| Season (spring 2009) | 1.62 | 0.56 | 2.88 | 0.005 |
| Body weight | 0.04 | 0.02 | 1.99 | 0.049 |
| *Il1b* expression |  |  |  |  |
| Intercept | -2.27 | 1.45 | -1.57 | 0.119 |
| Season (summer 2008) | -1.07 | 0.37 | -2.86 | 0.005 |
| Season (autumn 2008) | -1.38 | 0.40 | -3.45 | <0.001 |
| Season (winter 2008) | 0.17 | 0.42 | 0.40 | 0.689 |
| Season (spring 2009) | -0.49 | 0.55 | -0.89 | 0.37 |
| Body weight | 0.13 | 0.06 | 2.34 | 0.020 |
| Eye lens weight | 651.67 | 314.19 | 2.07 | 0.039 |
| Body weight × eye lens weight | -29.83 | 11.93 | -2.50 | 0.013 |
| *Il2* expression |  |  |  |  |
| Intercept | -0.18 | 0.30 | -0.60 | 0.550 |
| Season (summer 2008) | -0.25 | 0.44 | -0.56 | 0.576 |
| Season (autumn 2008) | -0.18 | 0.39 | -0.47 | 0.638 |
| Season (winter 2008) | 0.65 | 0.38 | 1.72 | 0.087 |
| Season (spring 2009) | 1.56 | 0.59 | 2.62 | 0.009 |
| *Il10* expression |  |  |  |  |
| Null | - | - | - | - |
| *Irf5* expression |  |  |  |  |
| Null | - | - | - | - |
| *Tbx21* expression |  |  |  |  |
| Intercept | -0.65 | 0.30 | -2.19 | 0.030 |
| Site (SQC) | 0.45 | 0.24 | 1.91 | 0.058 |
| Season (summer 2008) | -0.31 | 0.36 | 0.88 | 0.379 |
| Season (autumn 2008) | -1.02 | 0.32 | -3.22 | 0.005 |
| Season (winter 2008) | 1.23 | 0.31 | 3.96 | <0.001 |
| Season (spring 2009) | 1.50 | 0.48 | 3.10 | 0.002 |
| Sex (male) | 0.43 | 0.21 | 1.20 | 0.048 |
| *Tgfb1* expression |  |  |  |  |
| Intercept | -0.55 | 0.31 | -1.79 | 0.076 |
| Season (summer 2008) | -0.45 | 0.52 | -0.86 | 0.39 |
| Season (autumn 2008) | 0.28 | 0.43 | 0.65 | 0.51 |
| Season (winter 2008) | 1.07 | 0.39 | 2.77 | 0.006 |
| Season (spring 2009) | 1.15 | 0.84 | 1.37 | 0.174 |
| Sex (male) | 0.78 | 0.41 | 1.87 | 0.063 |
| Season (summer 2008) × sex (male) | -0.39 | 0.66 | -0.59 | 0.555 |
| Season (autumn 2008) × sex (male) | -0.07 | 0.56 | -0.13 | 0.900 |
| Season (winter 2008) × sex (male) | -1.38 | 0.52 | -2.63 | 0.010 |
| Season (spring 2009) × sex (male) | -0.59 | 1.10 | -0.53 | 0.592 |

^a^ *Gata3* expression measured at 96 h from splenocyte cultures stimulated with the mitogen PHA.

Deletion testing led to the removal of all non-genetic terms in the models of *Il10* and *Irf5* expression.
