# Supplementary material for: Genetic Diversity in Cytokines Associated with Immune Variation and Resistance to Multiple Pathogens in a Natural Rodent Population
Source: PLoS Genet. 2011 Oct 20;7(10):e1002343. doi: 10.1371/journal.pgen.1002343 (PMC3197692; doi:10.1371/journal.pgen.1002343)
Supplement: Table S3 — Post-hoc single SNP associations with immune profile. (DOC) [file pgen.1002343.s003.doc]

Table S3 Post-hoc single SNP associations with immune profile

| **SNP^a^** | **Response** | **Term^b^** | **Coefficient (s.e.)** | ***p*-value** |
| --- | --- | --- | --- | --- |
| *Il1b* 243 G/A (syn) | *Il1b* expression | A | 28.36 (11.86) | 0.074 |
|  |  | Heterozygote | -27.81 (11.92) | 0.166 |
|  | *Gata3* 96 h expression | A | -1039.98 (523.14) | 0.734 |
|  |  | Heterozygote | 1027.68 (527.21) | 0.230 |
| *Il1b* 253 A/G (nonsyn) | *Il1b* expression | G | -29.96 (11.80) | 0.131 |
|  |  | Heterozygote | -30.13 (12.04) | 0.450 |
|  | *Gata3* 96 h expression | G | 1104.40 (539.36) | 0.492 |
|  |  | Heterozygote | 1085.19 (534.70) | 0.634 |
| *Il1b* 324 C/T (syn) | *Il1b* expression | T | -28.51 (11.88) | 0.001 |
|  |  | Heterozygote | -29.51 (11.91) | 0.001 |
|  | *Gata3* 96 h expression | T | 981.35 (520.71) | 0.396 |
|  |  | Heterozygote | 1004.40 (529.53) | 0.160 |
| *Il2* 381 A/T (syn) | *Il10* expression | T | -0.08 (0.19) | 0.660 |
|  |  | Heterozygote | -0.25 (0.21) | 0.236 |
| *Il2* 408 C/G (nonsyn) | *Il10* expression | G | 0.09 (0.15) | 0.536 |
|  |  | Heterozygote | -0.27 (0.21) | 0.190 |
| *Il12b* 278 G/C (nonsyn) | *Il1b* expression | C | -29.46 (11.89) | 0.123 |
|  |  | Heterozygote | -29.46 (11.89) | 0.123 |
|  | *Il2* expression | C | -1.17 (0.45) | 0.009 |
|  |  | Heterozygote | -1.17 (0.45) | 0.009 |
| *Il12b* 704 C/T (nonsyn) | *Il1b* expression | T | -26.86 (11.82) | 0.002 |
|  |  | Heterozygote | -26.86 (11.82) | 0.002 |
|  | *Il2* expression | T | -1.06 (0.57) | 0.061 |
|  |  | Heterozygote | -1.06 (0.57) | 0.061 |

^a^ SNP designation includes the encompassing gene, SNP position relative to aligned mouse cDNA sequence and the resulting base change. (syn) and (nonsyn) relate to whether the substitution leads to a synonymous or nonsynonymous change in the translated protein.

^b^ Genetic terms were fitted either (i) under a heterozygote model where, for each SNP locus, values of heterozygotes were compared to homozygotes or (ii) an additive model, where the effect, *α_i_*, of the minor allele *a* relative to the more common allele *A* is assumed to be 0, *α_i_* and 2*α_i_* for genotypes *AA*, *Aa* and *aa*, respectively.
