# Supplementary material for: Genetic Diversity in Cytokines Associated with Immune Variation and Resistance to Multiple Pathogens in a Natural Rodent Population
Source: PLoS Genet. 2011 Oct 20;7(10):e1002343. doi: 10.1371/journal.pgen.1002343 (PMC3197692; doi:10.1371/journal.pgen.1002343)
Supplement: Table S4 — GLMs describing non-genetic factors associated with the probability of pathogen infection. (DOC) [file pgen.1002343.s004.doc]

**Table S4** GLMs describing non-genetic factors associated with the probability of pathogen infection

| **Term** | **Coefficient** | **s.e** | ***z*-value** | ***p*-value** |
| --- | --- | --- | --- | --- |
| *Nematode infection* |  |  |  |  |
| Intercept | -0.70 | 0.40 | -1.72 | 0.085 |
| Site (SQC) | -0.71 | 0.31 | -2.30 | 0.021 |
| Season (summer 2008) | -0.47 | 0.42 | -1.12 | 0.265 |
| Season (autumn 2008) | -1.34 | 0.42 | -3.22 | 0.001 |
| Season (winter 2008) | -1.30 | 0.44 | -2.98 | 0.003 |
| Season (spring 2009) | -1.53 | 0.82 | -1.87 | 0.061 |
| Sex (male) | 0.82 | 0.36 | 2.29 | 0.022 |
| *Cestode infection* |  |  |  |  |
| Intercept | 2.83 | 1.61 | 1.75 | 0.080 |
| Season (summer 2008) | -3.14 | 2.15 | -1.46 | 0.144 |
| Season (autumn 2008) | -4.89 | 1.79 | -2.73 | 0.006 |
| Season (winter 2008) | 1.14 | 2.31 | 0.49 | 0.623 |
| Season (spring 2009) | -0.08 | 3.07 | -0.03 | 0.979 |
| Body weight | 0.06 | 0.04 | 1.66 | 0.097 |
| Eye lens weight | -999.34 | 255.92 | -3.91 | <0.0001 |
| Sex (male) | 0.88 | 0.33 | 2.62 | 0.009 |
| Season (summer 2008) × body weight | 0.18 | 0.08 | 2.11 | 0.035 |
| Season (autumn 2008) × body weight | 0.16 | 0.07 | 2.19 | 0.028 |
| Season (winter 2008) × body weight | -0.20 | 0.12 | -1.76 | 0.078 |
| Season (spring 2009) × body weight | -0.07 | 0.15 | -0.48 | 0.631 |
| *Flea infection* |  |  |  |  |
| Intercept | -0.27 | 1.10 | -0.25 | 0.807 |
| Site (SQC) | -1.14 | 0.60 | -1.89 | 0.058 |
| Season (summer 2008) | -3.10 | 1.91 | -1.62 | 0.105 |
| Season (autumn 2008) | -0.83 | 1.66 | -0.50 | 0.619 |
| Season (winter 2008) | 3.34 | 2.39 | 1.40 | 0.162 |
| Season (spring 2009) | 5.04 | 3.03 | 1.66 | 0.096 |
| Body weight | 0.04 | 0.04 | 1.14 | 0.256 |
| Site (SQC) × Season (summer 2008) | 1.59 | 0.97 | 1.63 | 0.103 |
| Site (SQC) × Season (autumn 2008) | 0.26 | 0.75 | 0.35 | 0.724 |
| Site (SQC) × Season (winter 2008) | 2.82 | 0.85 | 3.30 | 0.001 |
| Site (SQC) × Season (spring 2009) | - | - | - | - |
| Season (summer 2008) × body weight | 0.12 | 0.07 | 1.77 | 0.077 |
| Season (autumn 2008) × body weight | 0.05 | 0.07 | 0.66 | 0.507 |
| Season (winter 2008) × body weight | -0.31 | 0.13 | -2.40 | 0.016 |
| Season (spring 2009) × body weight | -0.24 | 0.15 | -1.59 | 0.112 |
| *Tick infection* |  |  |  |  |
| Intercept | 2.25 | 2.76 | 0.82 | 0.415 |
| Season (summer 2008) | -6.66 | 3.07 | -2.17 | 0.030 |
| Season (autumn 2008) | -5.16 | 3.07 | -1.68 | 0.093 |
| Season (winter 2008) | -11.13 | 4.44 | -2.51 | 0.012 |
| Season (spring 2009) | -18.62 | 754.50 | -0.03 | 0.980 |
| Body weight | 0.09 | 0.03 | 2.93 | 0.003 |
| Eye lens weight | -1052.00 | 538.20 | -1.95 | 0.051 |
| Sex (male) | 0.35 | 0.75 | 0.47 | 0.637 |
| Season (summer 2008) × eye lens weight | 1809.00 | 628.70 | 2.88 | 0.004 |
| Season (autumn 2008) × eye lens weight | 676.90 | 647.10 | 1.05 | 0.296 |
| Season (winter 2008) × eye lens weight | 2506.00 | 1153.00 | 2.17 | 0.030 |
| Season (spring 2009) × eye lens weight | 530.40 | 1464.00 | 0.36 | 0.717 |
| Season (summer 2008) × sex (male) | -1.73 | 1.16 | -1.50 | 0.134 |
| Season (autumn 2008) × sex (male) | 0.88 | 1.02 | 0.86 | 0.390 |
| Season (winter 2008) × sex (male) | -0.59 | 1.21 | -0.49 | 0.627 |
| Season (spring 2009) × sex (male) | 15.67 | 754.50 | 0.02 | 0.983 |
| Babesia microti *infection* |  |  |  |  |
| Intercept | -2.56 | 0.76 | -3.35 | 0.001 |
| Season (summer 2008) | -1.14 | 0.51 | -2.23 | 0.026 |
| Season (autumn 2008) | 0.41 | 0.44 | 0.93 | 0.352 |
| Season (winter 2008) | 0.08 | 0.49 | 0.16 | 0.872 |
| Season (spring 2009) | -1.05 | 0.83 | -1.27 | 0.205 |
| Body weight | 0.06 | 0.02 | 2.48 | 0.013 |
| Bartonella *infection* |  |  |  |  |
| Intercept | -5.27 | 4.25 | -1.24 | 0.215 |
| Site (SQC) | 0.92 | 0.36 | 2.55 | 0.011 |
| Season (summer 2008) | 5.72 | 4.39 | 1.30 | 0.192 |
| Season (autumn 2008) | 2.25 | 4.49 | 0.50 | 0.616 |
| Season (winter 2008) | 7.91 | 4.85 | 1.63 | 0.103 |
| Season (spring 2009) | -1.71 | 5.61 | -0.30 | 0.761 |
| Eye lens weight | 517.87 | 780.52 | 0.66 | 0.507 |
| Season (summer 2008) × eye lens weight | -957.80 | 823.86 | -1.16 | 0.245 |
| Season (autumn 2008) × eye lens weight | -302.78 | 897.27 | -0.34 | 0.736 |
| Season (winter 2008) × eye lens weight | -2312.87 | 1144.28 | -2.02 | 0.043 |
