# Supplementary material for: Genetic Diversity in Cytokines Associated with Immune Variation and Resistance to Multiple Pathogens in a Natural Rodent Population
Source: PLoS Genet. 2011 Oct 20;7(10):e1002343. doi: 10.1371/journal.pgen.1002343 (PMC3197692; doi:10.1371/journal.pgen.1002343)
Supplement: Table S5 — GLMs describing non-genetic factors associated with variation in pathogen burden. (DOC) [file pgen.1002343.s005.doc]

Table S5 GLMs describing non-genetic factors associated with variation in pathogen burden

| **Term** | **Coefficient** | **s.e** | ***t*-value** | ***p*-value** |
| --- | --- | --- | --- | --- |
| *Cestode burden* |  |  |  |  |
| Intercept | 0.40 | 1.32 | 0.31 | 0.760 |
| Site (SQC) | 1.27 | 0.82 | 1.55 | 0.123 |
| Season (summer 2008) | 2.56 | 0.92 | 2.78 | 0.006 |
| Season (autumn 2008) | -1.10 | 1.11 | -0.99 | 0.322 |
| Season (winter 2008) | 2.18 | 2.28 | 0.96 | 0.340 |
| Season (spring 2009) | -0.06 | 2.57 | -0.02 | 0.981 |
| Body weight | 0.01 | 0.03 | 0.52 | 0.601 |
| Eye lens weight | -477.49 | 259.84 | -1.84 | 0.067 |
| Sex (male) | -1.61 | 0.79 | -2.02 | 0.044 |
| Site (SQC) × Season (summer 2008) | -1.98 | 0.45 | -4.41 | <0.0001 |
| Site (SQC) × Season (autumn 2008) | -1.08 | 0.57 | -1.89 | 0.060 |
| Site (SQC) × Season (winter 2008) | -0.74 | 0.79 | -0.95 | 0.345 |
| Site (SQC) × Season (spring 2009) | - | - | - | - |
| Site (SQC) × body weight | 0.08 | 0.03 | 2.90 | 0.004 |
| Site (SQC) × eye lens weight | -555.25 | 180.88 | -3.07 | 0.002 |
| Season (summer 2008) × body weight | -0.02 | 0.03 | -0.77 | 0.444 |
| Season (autumn 2008) × body weight | 0.08 | 0.04 | 2.09 | 0.037 |
| Season (winter 2008) × body weight | -0.16 | 0.12 | -1.33 | 0.184 |
| Season (spring 2009) × body weight | -0.05 | 0.13 | -0.39 | 0.698 |
| Eye lens weight × sex (male) | 630.45 | 245.61 | 2.57 | 0.011 |
| *Flea burden* |  |  |  |  |
| Intercept | -0.98 | 0.37 | -2.65 | 0.009 |
| Site (SQC) | -0.47 | 0.15 | -3.16 | 0.002 |
| Season (summer 2008) | 0.85 | 0.21 | 4.01 | <0.0001 |
| Season (autumn 2008) | 0.63 | 0.24 | 2.66 | 0.008 |
| Season (winter 2008) | -0.54 | 0.32 | -1.69 | 0.093 |
| Season (spring 2009) | 0.31 | 0.39 | 0.79 | 0.432 |
| Body weight | 0.04 | 0.01 | 4.26 | <0.0001 |
| *Tick burden* |  |  |  |  |
| Intercept | -9.86 | 3.77 | -2.61 | 0.009 |
| Season (summer 2008) | -8.96 | 2.48 | -3.62 | <0.001 |
| Season (autumn 2008) | 2.51 | 4.35 | 0.58 | 0.564 |
| Season (winter 2008) | -1.15 | 12.51 | -0.09 | 0.927 |
| Season (spring 2009) | 1.88 | 6.90 | 0.27 | 0.785 |
| Body weight | 0.75 | 0.16 | 4.75 | <0.0001 |
| Eye lens weight | 715.57 | 734.52 | 0.97 | 0.331 |
| Season (summer 2008) × body weight | -0.27 | 0.07 | -4.19 | <0.0001 |
| Season (autumn 2008) × body weight | -0.42 | 0.16 | -2.67 | 0.008 |
| Season (winter 2008) × body weight | -0.58 | 0.59 | -0.98 | 0.329 |
| Season (spring 2009) × body weight | -0.42 | 0.27 | -1.55 | 0.122 |
| Season (summer 2008) × eye lens weight | 3450.54 | 577.21 | 5.98 | <0.0001 |
| Season (autumn 2008) × eye lens weight | 1061.32 | 973.41 | 1.09 | 0.276 |
| Season (winter 2008) × eye lens weight | 2667.78 | 2722.31 | 0.98 | 0.328 |
| Season (spring 2009) × eye lens weight | 1489.39 | 1421.70 | 1.05 | 0.296 |
| Body weight × eye lens weight | -97.97 | 27.82 | -3.52 | <0.0001 |
