# Supplementary material for: Genetic Diversity in Cytokines Associated with Immune Variation and Resistance to Multiple Pathogens in a Natural Rodent Population
Source: PLoS Genet. 2011 Oct 20;7(10):e1002343. doi: 10.1371/journal.pgen.1002343 (PMC3197692; doi:10.1371/journal.pgen.1002343)
Supplement: Table S6 — GLMMs describing non-genetic factors associated with variation in the probability of pathogen infection. (DOC) [file pgen.1002343.s006.doc]

Table S6 GLMMs describing non-genetic factors associated with variation in the probability of pathogen infection.

| **Term** | **Coefficient** | **s.e** | ***z*-value** | ***p*-value** | **ΔAIC** |
| --- | --- | --- | --- | --- | --- |
| *Flea infection.* |  |  |  |  |  |
| Intercept | -1.47 | 0.56 | -2.63 | 0.008 | - |
| Site (SQC) | 0.51 | 0.40 | 1.30 | 0.194 | - |
| Season (summer 2008) | 1.94 | 0.53 | 3.63 | 0.0002 | 4.3 |
| Season (autumn 2008) | 1.01 | 0.56 | 1.79 | 0.072 | 4.3 |
| Season (winter 2008) | 0.19 | 0.89 | 0.217 | 0.829 | 4.3 |
| Sex (male) | 0.722 | 0.334 | 2.16 | 0.031 | - |
| Body weight | -0.003 | 0.019 | -0.21 | 0.835 | 40.1 |
| Site (SQC) × sex (male) | -1.05 | 0.438 | -2.40 | 0.016 | 3.4 |
| Random effect: *site*session* | σ^2^ = 0.36 ; sd = 0.60 |  |  |  |  |
| Random effect: *individual* | σ^2^ = 0.42 ; sd = 0.65 |  |  |  |  |
| *Tick infection* |  |  |  |  |  |
| Intercept | -1.71 | 0.39 | -4.40 | >0.0001 | - |
| Body weight | 0.014 | 0.017 | 0.818 | 0.413 | 28.2 |
| Random effect: *site*session* | σ^2^ = 0.07 ; sd = 0.26 |  |  |  |  |
| Random effect: *individual* | σ^2^ = 0.22 ; sd = 0.48 |  |  |  |  |
| Babesia microti *infection* |  |  |  |  |  |
| Intercept | -1.58 | 0.39 | -4.09 | >0.0001 | - |
| Site (SQC) | 0.42 | 0.48 | 0.88 | 0.378 | - |
| Season (summer 2008) | 0.58 | 0.48 | 1.21 | 0.225 | - |
| Season (autumn 2008) | 0.68 | 0.44 | 1.53 | 0.125 | - |
| Season (winter 2008) | 0.13 | 0.77 | 0.17 | 0.869 | - |
| Sex (male) | -0.70 | 0.34 | -2.07 | 0.038 | - |
| Body weight | 0.03 | 0.02 | 1.74 | 0.082 | 19.6 |
| Recapture (yes) | 0.56 | 0.23 | 2.41 | 0.016 | 3.8 |
| Site (SQC) × season (summer 2008) | -1.60 | 0.60 | -2.68 | 0.007 | 3.5 |
| Site (SQC) × season (autumn 2008) | -0.87 | 0.53 | -1.63 | 0.102 | 3.5 |
| Site (SQC) × season (winter 2008) | -1.66 | 1.09 | -1.52 | 0.128 | 3.5 |
| Site (SQC) × sex (male) | 1.30 | 0.44 | 2.96 | 0.003 | 5.4 |
| Random effect: *site*session* | σ^2^ = 0.00 ; sd = 0.00 |  |  |  |  |
| Bartonella *infection* |  |  |  |  |  |
| Intercept | 0.13 | 0.26 | 0.51 | 0.612 | - |
| Season (summer 2008) | 0.80 | 0.32 | 2.47 | 0.013 | 12.5 |
| Season (autumn 2008) | -0.45 | 0.34 | -1.33 | 0.182 | 12.5 |
| Season (winter 2008) | -2.51 | 0.74 | -3.39 | 0.0006 | 12.5 |
| Sex (male) | -0.22 | 0.21 | -1.04 | 0.297 | - |
| Body weight | -0.04 | 0.02 | -1.73 | 0.083 | - |
| Sex (male) × body weight | 0.06 | 0.03 | 2.13 | 0.03 | 3.5 |
| Random effect: *site*session* | σ^2^ = 0.01 ; sd = 0.10 |  |  |  |  |
| Random effect: *individual* | σ^2^ = 0.39 ; sd = 0.62 |  |  |  |  |

ΔAIC = the change in the AIC if the single term is dropped. σ^2^ = the variance attributable to a random effect. sd = standard deviation of σ^2^.
