# Supplementary material for: Genetic Diversity in Cytokines Associated with Immune Variation and Resistance to Multiple Pathogens in a Natural Rodent Population
Source: PLoS Genet. 2011 Oct 20;7(10):e1002343. doi: 10.1371/journal.pgen.1002343 (PMC3197692; doi:10.1371/journal.pgen.1002343)
Supplement: Table S7 — GLMMs describing non-genetic factors associated with variation in tick burden. (DOC) [file pgen.1002343.s007.doc]

Table S7 GLMMs describing non-genetic factors associated with variation in tick burden.

| **Term** | **Coefficient** | **s.e** | ***t*-value** | **ΔAIC** |
| --- | --- | --- | --- | --- |
| Intercept | -5.48 | 0.029 | -191.88 | - |
| Season (summer 2008) | 3.05 | 0.03 | 106.62 | - |
| Season (autumn 2008) | 0.76 | 0.03 | 24.61 | - |
| Season (winter 2008) | -0.57 | 0.06 | -10.42 | - |
| Sex (male) | 0.80 | 0.02 | 49.69 | - |
| Mature (yes) | 1.76 | 0.03 | 63.56 | - |
| Body weight | -0.15 | 0.00 | -62.23 | - |
| Season (summer 2008) × mature (yes) | -2.56 | 0.03 | -83.50 | 7.7 |
| Season (autumn 2008) × mature (yes) | -0.69 | 0.04 | -19.51 | 7.7 |
| Season (winter 2008) × mature (yes) | -10.70 | 17.57 | -0.61 | 7.7 |
| Sex (male) × body weight | 0.14 | 0.00 | 95.46 | 9.7 |
| Mature (yes) × body weight | 0.15 | 0.00 | 64.59 | 5.2 |
| Random effect: *site*session* | σ^2^ = 0.00 ; sd = 0.06 |  |  |  |
| Random effect: *individual* | σ^2^ = 0.01 ; sd = 0.08 |  |  |  |

ΔAIC = the change in the AIC if the single term is dropped. σ^2^ = the variance attributable to a random effect. sd = standard deviation of σ^2^
