# Supplementary material for: Genetic Diversity in Cytokines Associated with Immune Variation and Resistance to Multiple Pathogens in a Natural Rodent Population
Source: PLoS Genet. 2011 Oct 20;7(10):e1002343. doi: 10.1371/journal.pgen.1002343 (PMC3197692; doi:10.1371/journal.pgen.1002343)
Supplement: Table S8 — Post-hoc single SNP associations with pathogen resistance. (DOC) [file pgen.1002343.s008.doc]

**Table S8** Post-hoc single SNP associations with pathogen resistance

| **SNP^a^** | **Response^b^** | **Term^c^** | **Coefficient (s.e.)** | **p-value** | **ΔAIC^d^** |
| --- | --- | --- | --- | --- | --- |
| *Il1b* 243 G/A (syn) | Tick infection (CS) | A | 15.81 (748.42) | 0.773 | - |
|  |  | Heterozygote | 15.72 (750.25) | 0.180 | - |
|  | Bartonella infection (Long.) | A | 0.23 (0.21) | 0.246* | -0.8 |
|  |  | Heterozygote | -0.09 (0.25) | 0.837* | -2.0 |
|  | Flea infection (Long.) | A | -0.32 (0.22) | 0.137* | 0.0 |
|  |  | Heterozygote | -0.61 (0.27) | 0.026* | 2.8 |
| *Il1b* 253 A/G (nonsyn) | Tick infection (CS) | G | 15.68 (754.02) | 0.739 | - |
|  |  | Heterozygote | 15.97 (729.73) | 0.002 | - |
|  | Bartonella infection (Long.) | G | 0.22 (0.16) | 0.190* | -0.1 |
|  |  | Heterozygote | -0.02 (0.21) | 0.760* | -2.0 |
|  | Flea infection (Long.) | A | 0.50 (0.17) | 0.004* | 6.3 |
|  |  | Heterozygote | 0.14 (0.22) | 0.541* | -1.6 |
| *Il1b* 324 C/T (syn) | Tick infection (CS) | T | 15.80 (749.32) | 0.096 | - |
|  |  | Heterozygote | 15.72 (751.11) | 0.172 | - |
|  | Bartonella infection (Long.) | T | -0.34 (0.17) | 0.056* | 1.5 |
|  |  | Heterozygote | -0.46 (0.22) | 0.042* | 2.1 |
|  | Flea infection (Long.) | T | -0.26 (0.18) | 0.230* | -0.1 |
|  |  | Heterozygote | -0.42 (0.23) | 0.059* | 1.1 |
| *Il2* 381 A/T (syn) | Nematode infection (CS) | T | 0.70 (0.28) | 0.015 | - |
|  |  | Heterozygote | 0.87 (0.32) | 0.006 | - |
|  | Cestode burden (CS) | T | 648.32 (243.58) | 0.037 | - |
|  |  | Heterozygote | 646.91 (243.33) | 0.018 | - |
|  | Flea burden (CS) | T | -0.023 (0.14) | 0.857 | - |
|  |  | Heterozygote | -0.06 (0.15) | 0.708 | - |
|  | Tick infection (Long.) | T | -0.75 (0.33) | 0.015* | 3.2 |
|  |  | Heterozygote | -0.71 (0.34) | 0.023* | 2.5 |
|  | Tick burden (CS) | T | -82.86 (26.19) | 0.032 | - |
|  |  | Heterozygote | -87.74 (27.77) | 0.003 | - |
|  | Babesia infection (Long.) | T | 0.33 (0.24) | 0.173* | -0.2 |
|  |  | Heterozygote | 0.46 (0.25) | 0.074* | 1.3 |
|  | Bartonella infection (Long.) | T | -0.31 (0.24) | 0.087* | -0.4 |
|  |  | Heterozygote | -0.40 (0.25) | 0.051* | 0.4 |
| *Il2* 408 C/G (nonsyn) | Nematode infection (CS) | G | -0.19 (0.25) | 0.445 | - |
|  |  | Heterozygote | 0.11 (0.31) | 0.737 | - |
|  | Cestode burden (CS) | G | 633.74 (249.35) | 0.903 | - |
|  |  | Heterozygote | 622.31 (245.50) | 0.062 | - |
|  | Flea burden (CS) | G | -0.17 (0.12) | 0.141 | - |
|  |  | Heterozygote | -0.32 (0.155) | 0.034 | - |
|  | Tick infection (Long.) | G | 0.32 (0.19) | 0.100* | 0.6 |
|  |  | Heterozygote | -0.01 (0.26) | 0.955 | -2.0 |
|  | Tick burden (CS) | G | -96.56 (27.33) | 0.235 | - |
|  |  | Heterozygote | -96.08 (27.12) | 0.394 | - |
|  | Babesia infection (Long.) | G | 0.14 (0.16) | 0.380* | -1.2 |
|  |  | Heterozygote | 0.42 (0.22) | 0.047* | 1.6 |
|  | Bartonella infection (Long.) | G | -0.15 (0.16) | 0.434* | -1.2 |
|  |  | Heterozygote | -0.38 (0.21) | 0.077* | 1.1 |
| *Il12b* 278 G/C (nonsyn) | Nematode infection (CS) | C | 1.41 (0.44) | 0.002 | - |
|  |  | Heterozygote | 1.41 (0.44) | 0.002 | - |
|  | Babesia infection (Long.) | C | 1.06 (0.31) | <0.001* | 9.7 |
|  |  | Heterozygote | 0.75 (0.36) | 0.048* | 2.1 |
| *Il12b* 704 C/T (nonsyn) | Nematode infection (CS) | T | 0.28 (0.75) | 0.713 | - |
|  |  | Heterozygote | 0.28 (0.75) | 0.713 | - |
|  | Babesia infection (Long.) | T | 0.14 (0.77) | 0.869* | -2.0 |
|  |  | Heterozygote | 0.36 (0.86) | 0.667* | -1.8 |
| *Slc11a1* 537 C/G (nonsyn) | Flea burden (CS) | G | -0.12 (0.11) | 0.307 | - |
|  |  | Heterozygote | -0.25 (0.14) | 0.076 | - |
|  | Tick burden (CS) | G | -103.84 (32.05) | 0.495 | - |
|  |  | Heterozygote | -88.13 (26.92) | 0.013 | - |
| *Slc11a1* 714 G/A (syn) | Flea burden (CS) | A | -0.16 (0.11) | 0.154 | - |
|  |  | Heterozygote | -0.27 (0.14) | 0.055 | - |
|  | Tick burden (CS) | A | 109.54 (36.14) | 0.559 | - |
|  |  | Heterozygote | -91.61 (29.04) | 0.017 | - |
| *Tlr2* 1383 G/A (syn) | Cestode burden (CS) | A | -628.25 (246.39) | 0.876 | - |
|  |  | Heterozygote | 622.93 (245.85) | 0.485 | - |
| *Tlr2* 1648 G/A (nonsyn) | Cestode burden (CS) | A | -574.44 (249.03) | 0.529 | - |
|  |  | Heterozygote | 575.56 (247.530 | 0.453 | - |
| *Tlr2* 1706 G/A (nonsyn) | Cestode burden (CS) | A | 627.86 (247.09) | 0.242 | - |
|  |  | Heterozygote | 627.86 (247.09) | 0.242 | - |
| *Tlr2* 1944 T/C (syn) | Cestode burden (CS) | C | -587.74 (241.10) | 0.184 | - |
|  |  | Heterozygote | 636.39 (249.10) | 0.002 | - |
| *Tnf* 210 T/C (syn) | Cestode burden (CS) | C | 579.81 (245.18) | 0.238 | - |
|  |  | Heterozygote | 638.73 (245.64) | 0.666 | - |

^a^ SNP designation includes the encompassing gene, SNP position relative to aligned mouse cDNA sequence and the resulting base change. (syn) and (nonsyn) relate to whether the substitution leads to a synonymous or nonsynonymous change in the translated protein.

^b^ ‘Infection’ responses refer to probability of infection while ‘burden’ refers to parasite load. Data were sourced from either the cross-sectional (CS) of longitudinal (Long.) studies.

^c^ Genetic terms were fitted either (i) under a heterozygote model where, for each SNP locus, values of heterozygotes were compared to homozygotes or (ii) an additive model, where the effect, *α_i_*, of the minor allele *a* relative to the more common allele *A* is assumed to be 0, *α_i_* and 2*α_i_* for genotypes *AA*, *Aa* and *aa*, respectively.

^d^ Change in the AIC if the single term is dropped.

* *P*-values for analyses using GLMMs are for equivalent GLMs.
