# Supplementary material for: Genetic Diversity in Cytokines Associated with Immune Variation and Resistance to Multiple Pathogens in a Natural Rodent Population
Source: PLoS Genet. 2011 Oct 20;7(10):e1002343. doi: 10.1371/journal.pgen.1002343 (PMC3197692; doi:10.1371/journal.pgen.1002343)
Supplement: Table S9 — Primers and PCR conditions used to amplify gene products from cDNA. (DOC) [file pgen.1002343.s009.doc]

Table S9 Primers and PCR conditions used to amplify gene products from cDNA.

| **Gene** | **Primers** | **PCR *T*_a_ (°C)** | **Product size (bp)** |
| --- | --- | --- | --- |
| *Ifng* | For: GAGCCARATTRTCTCTTTCTAC | 50.8 | 250 |
|  | Rev: GACTCCTTTTCCGCTTCC |  |  |
| *Il1b* | For: ATTGTRGCTKTGGAGAAGCTG | 53.2 | 470 |
|  | Rev: CTTGWGAGGTGCTGATGTACC |  |  |
|  | For2: CAAGTGTCTGAAGCAGCYATG | 48.0 | 370 |
|  | Rev2: CTGACGAATGGGAACATC |  |  |
| *Il2* | For: ARCAGYGCACCYACTTCAAG | 55.4 | 390 |
|  | Rev: TGYTGAGATGATGCTTTGAC |  |  |
| *Il5* | For: CAKTGGTGAAAGAGACCTTG | 53 | 290 |
|  | Rev: AACTCTTGCAGGTARTCTAGG |  |  |
| *Il10* | For: TGCCAAGCCTTRTCKGARATG | 54.3 | 250 |
|  | Rev: GGTTGATGAAGATGTCAAAYT |  |  |
| *Il12b* | For: AGATGCTGGCCARTACACC | 58.6 | 540 |
|  | Rev: AGGGAGAAGTAGGAATGKGGAG |  |  |
| *Il18* | For: TGGAATCAGACMACTTTGGC | 59.2 | 440 |
|  | Rev: GATTTATCCCCATTTTCATCC |  |  |
| *Slc11a1* | For: ATCCTCCTCTGGCTGACC | 58.2 | 1060 |
|  | Rev: GGAGGCTGGGCAGGTAG |  |  |
| *Tgfb1* | For: CATCGARGCCATCCGNGG | 57.9 | 950 |
|  | Rev: CGTAGTACACGATGGGCAG |  |  |
| *Tlr2* | For: GYGAAAATYTGATGGTTGAAG | 50.0 | 1020 |
|  | Rev: AGAAGTCCAGTTCRTACTTGC |  |  |
| *Tlr4* | For: AAATGGCTGGCAATTCTTTC | 58.0 | 1000 |
|  | Rev: AGTCKTCTCCAGAAGATGTGC |  |  |
| *Tnf* | For: ACCATGAGCACAGAAAGCAT | 55.4 | 600 |
|  | Rev: CTTCTCCAGCTGGAAGACT |  |  |

PCRs were performed in 20 µl reactions comprising of 1 µl 1:10 vole cDNA, 1 µl each of 10 µM forward and reverse primers, 10 µl Biomix Red PCR ready mix (Bioline) and 7 µl water. PCR conditions were as follows: 95 °C for five minutes, followed by 35 cycles of 95 °C for 30 seconds, various *T*_a_ for 30 seconds, and 72 °C for one minute, with a final extension time of 72 °C for seven minutes. Nucleotide key: A = adenine; C = cytosine; G = guanine; T = thymine; M = A or C; R =A or G; W = A or T; Y = C or T; K = G or T.
