# Supplementary material for: Genetic Diversity in Cytokines Associated with Immune Variation and Resistance to Multiple Pathogens in a Natural Rodent Population
Source: PLoS Genet. 2011 Oct 20;7(10):e1002343. doi: 10.1371/journal.pgen.1002343 (PMC3197692; doi:10.1371/journal.pgen.1002343)
Supplement: Table S10 — Haplotype frequencies inferred from genotyping data for the combined cross-sectional and longitudinal datasets. (DOC) [file pgen.1002343.s010.doc]

**Table S10** Haplotype frequencies inferred from genotyping data for the combined cross-sectional and longitudinal datasets.

| **Site** | **BLB** | | | **SQC** | | | **All** | | |
| --- | --- | --- | --- | --- | --- | --- | --- | --- | --- |
| **Gene** | ***2n*** | **Haplotype** | **Frequency** | ***2n*** | **Haplotype** | **Frequency** | ***2n*** | **Haplotype** | **Frequency** |
| *Il1b* | 466 | GAC | 0.36 | 660 | GGC | 0.36 | 1126 | GAC | 0.32 |
|  |  | GGC | 0.27 |  | GAC | 0.29 |  | GGC | 0.32 |
|  |  | GAT | 0.23 |  | GAT | 0.21 |  | GAT | 0.22 |
|  |  | AAC | 0.14 |  | AAC | 0.13 |  | AAC | 0.13 |
|  |  | GGT | <0.01 |  | GGT | 0.01 |  | GGT | 0.01 |
| *Il2* | 468 | AC | 0.53 | 644 | AC | 0.55 | 1112 | AC | 0.54 |
|  |  | AG | 0.37 |  | AG | 0.32 |  | AG | 0.34 |
|  |  | TC | 0.10 |  | TC | 0.13 |  | TC | 0.11 |
| *Il12b* | 475 | GC | 0.90 | 653 | GC | 0.97 | 1128 | GC | 0.94 |
|  |  | CC | 0.03 |  | CC | 0.03 |  | CC | 0.04 |
|  |  | GT | 0.07 |  | - |  |  | GT | 0.01 |
| *Slc11a1* | 480 | CG | 0.65 | 658 | 488 | 0.74 | 1138 | CG | 0.70 |
|  |  | GA | 0.32 |  | 152 | 0.23 |  | GA | 0.27 |
|  |  | GG | 0.02 |  | 18 | 0.03 |  | CA | 0.02 |
|  |  | CA | 0.01 |  | - | - |  | GG | 0.01 |
| *Tgfb1* | 454 | C | 0.65 | 650 | C | 0.77 | 1104 | C | 0.72 |
|  |  | A | 0.35 |  | A | 0.23 |  | A | 0.28 |
| *Tlr2* | 468 | GGGC | 0.56 | 660 | GGGC | 0.51 | 1128 | GGGC | 0.53 |
|  |  | GGGT | 0.28 |  | GGGT | 0.32 |  | GGGT | 0.30 |
|  |  | GAGC | 0.11 |  | GAGC | 0.11 |  | GAGC | 0.11 |
|  |  | AGGC | 0.05 |  | AGGC | 0.05 |  | AGGC | 0.05 |
|  |  | GAGT | 0.01 |  | - | - |  | GAGT | <0.01 |
| *Tlr4* | 458 | AGC | 0.92 | 648 | AGC | 0.95 | 1106 | AGC | 0.94 |
|  |  | GTA | 0.05 |  | GGA | 0.04 |  | GTA | 0.03 |
|  |  | GGA | 0.02 |  | GTA | 0.01 |  | GGA | 0.02 |
|  |  | GGC | 0.01 |  | - | - |  | GGC | <0.01 |
|  |  | AGA | 0.01 |  | - | - |  | AGA | <0.01 |
| *Tnf* | 470 | C | 0.60 | 650 | C | 0.62 | 1120 | C | 0.61 |
|  |  | A | 0.40 |  | A | 0.38 |  | A | 0.39 |
