# Supplementary material for: Genetic Diversity in Cytokines Associated with Immune Variation and Resistance to Multiple Pathogens in a Natural Rodent Population
Source: PLoS Genet. 2011 Oct 20;7(10):e1002343. doi: 10.1371/journal.pgen.1002343 (PMC3197692; doi:10.1371/journal.pgen.1002343)
Supplement: Table S11 — Summary of splenocyte culture assays and mRNA measurements taken. (DOC) [file pgen.1002343.s011.doc]

**Table S11** Summary of splenocyte culture assays and mRNA measurements taken.

|  | **24 h splenocyte assay** | | | **96 h splenocyte assay** | |
| --- | --- | --- | --- | --- | --- |
| **Gene** | **Unstim.** | **HKLM** | **Imiqui.** | **Unstim.** | **PHA** |
| *Il1b* | ✓ | ✓ | - | - | - |
| *Irf5* | ✓ | - | ✓ | - | - |
| *Il10* | ✓ | ✓ | ✓ | ✓ | ✓ |
| *Tgfb1* | ✓ | ✓ | - | ✓ | ✓ |
| *Ifng* | - | - | - | ✓ | ✓ |
| *Il2* |  |  |  | ✓ | ✓ |
| *Foxp3* | - | - | - | ✓ | ✓ |
| *Gata3* | - | - | - | ✓ | ✓ |
| *Tbx21* | - | - | - | ✓ | ✓ |

Unstim., unstimulated cell culture; HKLM, culture stimulated with the TLR2 agonist HKLM; Imiqui, culture stimulated with the TLR7 agonist imiquimod; PHA, culture stimulated with the mitogen PHA.
