# Supplementary material for: Genetic Diversity in Cytokines Associated with Immune Variation and Resistance to Multiple Pathogens in a Natural Rodent Population
Source: PLoS Genet. 2011 Oct 20;7(10):e1002343. doi: 10.1371/journal.pgen.1002343 (PMC3197692; doi:10.1371/journal.pgen.1002343)
Supplement: Table S12 — Immune response variables derived from combining multiple measurements. (DOC) [file pgen.1002343.s012.doc]

**Table S12 Immune response variables derived from combining multiple measurements.**

| ***Standardized variables*** | | | |
| --- | --- | --- | --- |
| **Response** | **Component** | ***r*** | ***p*** |
| *Foxp3* | 96 h expression | 0.215 | 0.005 |
|  | 96 h PHA-stimulated expression |  |  |
| *Ifng* | 96 h expression | 0.482 | <0.001 |
|  | 96 h PHA-stimulated expression |  |  |
| *Il1b* | 24 h expression |  |  |
|  | 24 h TLR2-stimulated expression | 0.384 | <0.001 |
| *Il2* | 96 h expression | 0.435 | <0.001 |
|  | 96 h PHA-stimulated expression |  |  |
| *Irf5* | 24 h expression | 0.395 | <0.001 |
|  | 24 h TLR7-stimulated expression |  |  |
| *Tbx21* | 96 h expression | 0.324 | <0.001 |
|  | 96 h PHA-stimulated expression |  |  |
| ***Principal components variables*** | | | |
| **Response** | **Component** | **Loading** | **Proportion** |
| *Il10*^PC1^ | 24 h expression | 0.498 | 0.38 |
|  | 24 h TLR2-stimulated expression | 0.553 |  |
|  | 24 h TLR7-stimulated expression | 0.616 |  |
|  | 96 h expression* | 0.059 |  |
| *Tgfb1^PC1^* | 24 h expression | 0.610 | 0.34 |
|  | 24 h TLR2-stimulated expression | 0.527 |  |
|  | 96 h expression | 0.305 |  |
|  | 96 h PHA-stimulated expression | 0.508 |  |

‘Standardized variables’ lists the correlation coefficient (*r*) and *p*-value between two measures of expression. ‘Principal components variables’ lists component loadings of individual variables for the first principal component of each response and the proportion of total variation explained by the dominant PC. Unstimulated and mitogen-stimulated 96 h *Gata3* expression were measured and analyzed separately (see Materials and Methods).
